# A scoping review of voluntary male mentoring: Themes to connect adult men

**DOI:** 10.1111/hsc.13906

**Published:** 2022-07-18

**Authors:** Mark Henderson, Mark Hughes, John Hurley, Gregory Smith

**Affiliations:** ^1^ Faculty of Health Southern Cross University Coffs Harbour New South Wales Australia

**Keywords:** community participation, connection, disconnection, male loneliness, mentor, volunteer

## Abstract

In contemporary western cultures, such as Australia, there is increasing reported social disconnection. This disconnection is associated with loneliness and for some suicide, particularly for men. Voluntary male mentoring organisations aim to socially connect and improve men's wellbeing through facilitating men's one‐to‐one relationships. As is the case with most people, men value someone with whom they may communicate openly and safely, as occurs in voluntary mentoring. However, there is limited research on voluntary male mentoring or the experiences of the participants. This scoping review of the literature from voluntary adult male mentoring thematically synthesises the reported narratives from mentors. A systematic search was undertaken of five academic databases on voluntary adult male mentoring for scholarly articles in English. Additionally, the returned articles had their references reviewed for relevant authoritative reports and publications. After duplicates were removed, there were 471 publications. Sources included publications from humanities, social science, psychology and the health sciences. Once screened for eligibility this number was reduced to 29 publications. A thematic synthesis of the literature found the concepts of intentional relationship, journey and mutual growth were present. The implications of these findings are that men in community settings, informed by the experience of voluntary mentors, may be prepared to engage with other men. Additional research on the lived experience of mentors may offer further insights into facilitating male connection.

**What is known about this topic**

- Some men in western societies are experiencing social disconnection and loneliness.
- Volunteers who form a connection with other men in their communities are sometimes described as mentors.
- Community organisations, under the banner of male mentoring, are emerging to offer men connection.

**What this paper adds**

- A systematic examination of the literature from adult male volunteer mentoring.
- A synthesis of the narratives in the literature that produces themes to inform community organisations and their participants in the area of male connection.
- Future research opportunities that explore the experience of active volunteer mentors are identified.

## 1 INTRODUCTION

In many western societies, social connectedness has been identified as a protective factor for men's health and wellbeing (Dinkins, 2017; Robinson et al., 2015). Yet many men, even when they recognise loneliness in themselves or others, are unsure how to address it (Arbes et al., 2014; Feo, 2013). Some community‐based voluntary male organisations operating under the banner of mentoring in Australia are connecting men in one‐to‐one relationships, claiming to improve men's wellbeing (MentoringMen, 2022; Mentors4Men, 2022). However, the current language and themes in the mentoring literature, which has overwhelmingly emerged from the corporate sector, appear to have limited relevance to these voluntary participants (Mate et al., 2019). This scoping review of the mentoring literature from volunteers who mentor other adult men aims to present a relevant synthesis of men's experiences of forming connection with other men, particularly for men in community setting in modern western societies.

The word “mentor” entered the English language within the erudite letters from Lord Chesterfield to his son, in which he requested his son to accept him as his “mentor” so “he may go far” (Chesterfield et al., 1900 Letter CCLXXIV). This term, mentor, was adopted from a central character in one of the most popular books of the 18th century written by Lord Fenelon (1715), Archbishop of Cambrai in 1699, known as “The Adventures of Telemachus, the Son of Ulysses” (de La Mothe & Smollett, 1997). The author had re‐written a part of the Greek fable “The Odyssey” by Homer (circa BCE 800), giving mentor the abilities of sage wisdom and educational counsel with which to serve the young prince Telemachus on his voyage.

Renewed interest in mentoring for adults was sparked in 20th century western culture by qualitative American research investigating male adult development. Levinson (1978) interviewed 40 men from varied industries and backgrounds over several years with regard to their phases of life. Mentoring was frequently referred to as a necessary male adult development task for both mentor and mentee. Programs under the banner of mentoring formed in corporate organisations and educational institutions seeking performance improvement (Bozeman & Feeney, 2008). After three decades of mentoring research, largely embedded in corporate America, the following definition was posited as useful and is often cited:

A process for the informal transmission of knowledge, social capital, and psychosocial support perceived by the recipient as relevant to work, career, or professional development; mentoring entails informal communication, usually face‐to‐face and during a sustained period of time, between a person who is perceived to have greater relevant knowledge, wisdom, or experience (the mentor) and a person who is perceived to have less (the protégé). (Bozeman & Feeney, 2007, p. 14)

Corporate and educational mentoring was widely examined by private and public institutions with a primary focus on organisational performance and professional standing that connected mentoring to career progression and psychosocial support (Baird & Kram, 1983; Craig et al., 2013; Noe, 1988). Mentorship studies assessed the mentoring dyad considering age, race, gender and meeting frequency, as well as formal arrangement, duration, location and communication (Allen & Eby, 2008; Chao et al., 1992; Marshall‐Triner, 1986). The necessity of trust, respect, listening skills and purpose for the relationship were consistently noted as valuable to mentorship formation, with both mentor and mentee perceiving benefits from the mentoring partnership (Appelbaum et al., 1994; Arora & Rangnekar, 2015; Awaya et al., 2003; Lucas, 2001). A variety of quantitative scales were developed to evaluate these mentorship programs (Clutterbuck, 1991; Klasen, 2002). Scales included indicators such as directive—non‐directive, stretching—nurturing, counselling—coaching and networking—guardianship and presented mentoring as inclusive of activities from a variety of sectors (Moorcroft, 2014). Nonetheless, much of the thinking, language and ideas in the area were directed to corporate objectives.

Men, as do all people, connect with each other in a variety of settings extraneous to corporate organisations. The key mechanisms of active listening and commitment that facilitate corporate mentoring (Ragins & Kram, 2007) also occur in many areas such as gerontology, social service and community care (Cheetham et al., 2018; Zucchero, 2010). Unsurprisingly, voluntary relationships that encompass care, advice and a desire to contribute are often identified by participants as mentoring (Greenwood & Habibi, 2014; Mahoney et al., 2020). To a greater extent, the intimacy and trust which underpins the psychosocial component of corporate mentoring (Bozeman & Feeney, 2007) are expansively described as warmth and nurturance in the volunteering literature (Smith & Greenwood, 2014; Webber, 2011). More so, mentors and community leaders report seeing beyond others' perceived differences to value and include others, when the mentorship extends beyond the bounds of an organisational identity (Wilson, Bigby, et al., 2013). Critically examining the mentoring literature inclusive of areas such as health, relationships and ageing may present insights into the utility of mentoring outside of career alone. Mentoring dyads that are entered into voluntarily by the participants may provide narratives relevant to social connection in community settings.

The plethora of literature involving mentorship, including the literature on volunteer mentoring, often evaluates the achievements and outcomes of the mentee (Cordier et al., 2016). Similarly, much of the literature focuses on adult to child mentoring and whilst the mentor may be a volunteer, the recipient may have little say in their role (Brady et al., 2017). There remains a lack of detail on voluntary adult mentoring in Australia from the mentor's perspective. The social disconnection experienced by men warrants a gender‐specific consideration of the mentoring literature (Arbes et al., 2014). An examination of the narratives for the themes present in voluntary adult male mentoring in western cultures may inform the practice of men connecting to other men.

## 2 METHODOLOGY

The lived experience of voluntary adult male mentors in western society was examined through a scoping review of the literature. This was conducted by constructing key themes from the narratives present in the systematically searched literature. A systematic search of scholarly publications in English, inclusive of all literature up to April 2021 was undertaken on the topic of voluntary male mentoring. The search included databases of peer‐reviewed journals, seminal authors from the mentoring literature, grey literature from Australian authoritative reports, and a manual search of relevant cited references to ensure the search was extensive. A thematic analysis was then conducted on the literature to contextualise the language and descriptions of men's lived experience of mentorship, which is presented in the findings.

**FIGURE 1:**
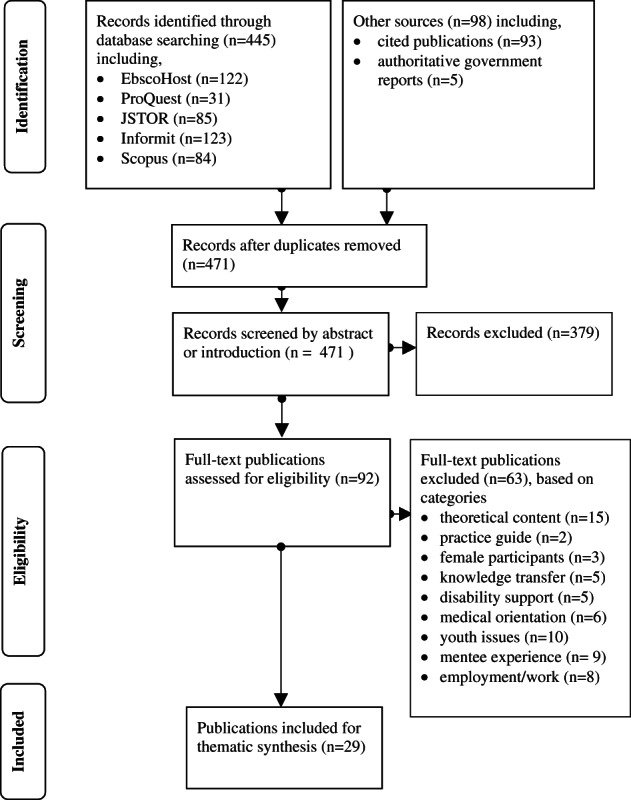
PRISMA flowchart

This review focused on voluntary adult male to male mentoring relationships. The specific search terms used were “mentor,” “volunteer” and “male” and its synonyms, and not “school.” School mentoring was excluded as the mentees are minors and not voluntary participants (DuBois et al., 2011; Sale et al., 2008). The sources were scholarly articles in English from academic databases across the humanities, social science, psychology and health collections. The databases searched were EbscoHost, ProQuest, JSTOR, Informit and Scopus and no time limit was set. The 445 articles returned from the database search had their citations screened for related terms that resulted in 98 publications being identified. Once duplicates were removed there were 471 publications which were screened by abstract or introductory summary. A total of 92 full‐text publications were then reviewed for eligibility. This process is shown diagrammatically in Figure 1.

This article reviews the mentoring literature with a focus on adult volunteers who mentor adult men. The aim is to present an evidenced narrative of mentoring applicable for utilisation by community participants with the potential to connect with men in modern western societies.

This review sought the interpreted narratives of participants who voluntarily connect with men, therefore publications were excluded from this review that did not examine the lived experience of mentors. A substantial body of the scholarly literature on volunteering contains the key terms of mentoring (Sanchez & Ferrari, 2005; Wilson, Cordier, et al., 2013), but much of the volunteering literature was excluded from this review as it was concerned with the mentor's evaluation of the mentee's success at learning or overcoming perceived shortcomings in areas such as knowledge transfer, youth issues, medical assistance or disability support (Ayton & Joss, 2016; Marks et al., 2019; Wingfield et al., 2017). Literature on mentoring in employment and work, whilst rich in content and providing a seminal definition of mentoring (Higgins & Kram, 2001), was excluded owing to the paid nature of the mentorship, lack of a clear voluntary commitment, and the focus on mentee performance within the organisation (de Vries et al., 2006). The research team met on multiple occasions and decided on the inclusion criteria. Articles were categorised as included, excluded or possible. The possible articles were discussed by all team members for suitability.

For inclusion in the review, the articles had to meet any one of the following criteria:

- Describe the adult male lived experience of voluntary mentoring.
- Identify adult male behaviour or a relationship involving mentorship.
- Contain adult male relationship narratives involving mentoring.
- Explore voluntary engagement which includes mentoring.

The literature included for thematic synthesis comprised 25 scholarly articles, two complete peer‐reviewed books, and two authoritative government reports. The earliest inclusion was 1978 and the latest April 2021. The selected literature reported on two quantitative, 14 qualitative and 13 mixed‐method studies. The literature covered a range of disciplines including sociology, psychology, medicine, nursing, leadership, management, education and gerontology. An Australian perspective was presented with both government reports and nine of the scholarly articles principally drawing on Australian research.

The final set of publications was assessed according to each of the four inclusion criteria, resulting in 19 publications meeting one criterion, five meeting two criteria, three meeting three criteria and two meeting all four criteria. Levinson's seminal book, ‘The Seasons of a Man's Life’ (Levinson, 1978), which pre‐empted the resurgent interest in formal mentoring programs, met all four of the inclusion criteria. Similarly, a large Australian mixed‐methods study commissioned by BeyondBlue and Movember, titled the ‘Men's social connectedness report’ (Arbes et al., 2014), also met all four inclusion criteria. This report contained rich content of adult men's lived experience inclusive of their narratives with regard to communicating with other men in their communities.

Each of the publications included for review was subjected to a thematic analysis to identify key narratives related to men's lived experience of voluntary mentoring. Transparency was applied with the use of specific key terms and an extensive search of the topic (Hannes & Macaitis, 2012). The strength of the thematic synthesis of a body of literature lies in its potential to form categories which are more accessible to the reader, based on common elements across otherwise heterogeneous studies (Dixon‐Woods et al., 2006). Qualitative research findings are usually stated in terms of themes or categories (Byrne, 2001). The included publications had their key narrative phrases and conclusions summarised from which labels were constructed. This approach to qualitative analysis of the literature was designed to interpret and represent the available textual data using a structured method (Nowell et al., 2017). As they relate to the labels from the literature, the thematic categories are then discussed.

Direct quotes and summary statements that encapsulated the mentors' lived experience from the selected literature were identified. These labels were grouped with consideration of similarity and meaning through an iterative process with frequent research team meetings. Key words were then identified that categorised the groupings and were titled Mentorship Category Themes. These themes were independently reviewed by the authors with reference to the included articles as an additional iterative process for theme refinement (Groenewald, 2004). This is presented in Table 1: Mentorship Category Themes.

**TABLE 1:** Mentorship Category Themes

Mentorship Category Themes	Labels identified from the literature
Intentional relationship	Contributing and giving back One to one, non‐judgmental active listening Care and communication
Journey	Transitional figures Paths crossing Passing the baton
Mutual growth	Authentic emotional learning Reciprocal and coherent Meaningful and Generative

## 3 FINDINGS

The three themes constructed from the literature were:

1. Intentional relationship,
2. Journey, and
3. Mutual growth.

These categories were analysed from specific narratives within the literature, which contain the experiences, practices and emergent products of voluntary mentoring from the mentors' perspective. Each label relates to a direct quote or summarises the mentors' experiences as presented in the literature.

The first theme of an intentional relationship recognised that mentoring required a relationship that was entered into with forethought and a purpose (Rahja et al., 2016). In the mentorship literature, it was apparent that intentional effort by participants was required (Bozeman & Feeney, 2008). Specifically, this comprised effort with determination as quoted in multiple articles: “to give something back” (Emlet & Harris, 2020, p. 6; Ragins & Kram, 2007, p. 132). Voluntary mentors self‐nominated and showed up, which are important factors in forming mentoring dyads (Noe, 1988). They engaged with formal induction and pairing processes which were usually facilitated. Additionally, they recognised their contribution as an inclusive process that resulted in feelings of shared success, with the potential to create a legacy (Celdrán et al., 2018). In the mentorship literature, the humanistic process of non‐judgmental active listening, which required intention on the part of the mentor, was essential to a relationship perceived as having depth, and hence being worthwhile (Greenwood & Habibi, 2014; Santini et al., 2020; Zucchero, 2010). “A good mentor listens” and does “not try to solve their problems” was a frequent sentiment (Straus et al., 2013, pp. 3, 5). Similarly, voluntary relationships with a preparedness to demonstrate care and actively listen were evident within mentoring to alleviate loneliness in community‐based caregivers and older people (Greenwood & Habibi, 2014; Zucchero, 2011). The notion of “befriending” was used by participants to refer to profound relationships forming in which the roles were blurred, and “life experience” was shared (Zucchero, 2010).

The second theme of the journey was reflective of the original mentor fable by Homer (circa 800 BCE) as the story commences with the sage advice to “journey” forth to seek the king (Butcher & Lang, 1890). The literature reports that transitional figures were present at the right place (Cheetham et al., 2018), and were “necessary for adult development” for both mentors and mentees (Levinson, 1978, p. 388). The eligible mentoring literature was peppered with terms and phrases associated with a journey such as “paths crossing” (Greenwood & Habibi, 2014) intergenerational meetings during “life course” (Zucchero, 2011), and “next steps” (Dennett, 2008). Many of the phrases suggest movement with regard to guidance through difficult times or emotional development (Wingfield et al., 2017). A direct quote from a volunteer in a mentoring capacity was “passing the baton”, providing an apt metaphor for handing over responsibility in a relay (Celdrán et al., 2018). The journey in this case was not one of physicality, but of the ideas and shared experiences which may arise between people when they spend time together.

The third theme of mutual growth was also apparent in the scholarly literature on volunteer mentoring. In an intergenerational volunteering situation, both parties spoke of preconceptions being replaced by the experience of “mutuality” (Zucchero, 2010, p. 15). Authenticity and emotional learning were described as unexpectedly occurring in volunteer relationships of depth, particularly in dimensions relating to self‐confidence and empathy, as social skills emerged (Shapira‐Lishchinsky & Levy‐Gazenfrantz, 2016). Interestingly, Levinson situated the mentor as “needing the recipient” of his mentoring just as much as the reverse, to ensure life transitions were fulfilled (Levinson, 1978, p. 253), with intergenerational learning described as leading to “developing a critically important and enduring attitude” (Santini et al., 2020, p. 16). Similarly, “helping others” was reported to be a feature of the masculine sense of coherence in Australian society by younger mentors (Adegbosin et al., 2019, p. 5) and “advice to others” was stated as a key mechanism in older adults' experience of “making life meaningful” (Schafer & Upenieks, 2016, p. 4). Mentors recognised the importance of reciprocity as shown by the following quote: “So, in my giving back in that area, that has been… in a way, it's kind of providing support to me, because it keeps me busy” (Emlet & Harris, 2020, p. 7). Men have identified mentorship as providing a positive strategy for maintaining physical and mental health (Proudfoot et al., 2015).

## 4 DISCUSSION

The social construct of voluntary adult male mentoring has received little examination in the academic literature. Adult mentoring with its association with corporate advancement has largely excluded the examination of unpaid volunteering, even though volunteering engenders many of the processes which facilitate mentorship. This scoping review of the literature on male volunteer mentoring has synthesised three themes from the narratives that may assist community participants to interpret their connection with other men.

An intentional relationship as occurring in voluntary male mentoring is usually one‐to‐one and involves a significant investment of time. Typically, this could be a weekly meeting of an hour which occurs over months or years. Importantly, in volunteer mentoring‐based relationships, transactional exchange is unexpected. The mentors' precept is to contribute, which in the initial stages usually requires them to show up and listen, thereby establishing some trust which underpins connection (Chun et al., 2010). The participants are present with each other for a period of time in which they expect to develop communication in some way (Butera, 2008). The mentor participant has some of the mentoring components in their awareness as presented in the narratives from the literature as “contribution”, “giving”, “active” and some aspect of being a “friend” (Cattan et al., 2005; Smith & Greenwood, 2014). These often positive and affirming ideas of being a man willing to support other men (Arbes et al., 2014) may embolden the participant conscious of an intentional relationship with the impetus to connect.

Journey, the second theme from the literature on voluntary male mentoring is descriptively present in the journey metaphor that emerges from person‐centred care in health services (Barker, 2001). Transitional by nature, a journey with care for travelling companions, aligns with Levinson's (1978) work which detailed a developmental approach for male adulthood phases throughout life. Levinson (1920‐1994) recognised, among other things, a necessary and formative adult relationship that he labelled mentoring that benefitted the participants. Often in adult volunteering, people from diverse backgrounds describe experiencing new perspectives as their paths cross in one‐to‐one interactions (Santini et al., 2020; Zucchero, 2011). Mentors have described participation in programs which facilitate the capacity building of others as meaningful and engaging, and as beneficial to their well‐being. (Chamravi et al., 2019; Cheetham et al., 2018; Yuen, 2002). A journey is usually finite or temporary, which as a theme for participants forming a connection to another person, implies limits on the relationship in terms of time and resources (Celdrán et al., 2018). Potential participants may have greater willingness to connect with unknown others if they have prior knowledge that their commitment would not be open‐ended.

The third theme of mutuality is strongly engendered in the voluntary community, particularly in adult male mentoring (Schafer & Upenieks, 2016; Shapira‐Lishchinsky & Levy‐Gazenfrantz, 2016). This is in part due to participants actively performing a peer‐led process of social connection and emotional support to other men. Both men connect within the mentoring dyad and are afforded the opportunity to practice social skills and possibly help one another (Adegbosin et al., 2019). Many men experience reciprocity in Australian society, which when enacted well is typified by being good‐natured, friendly and highly appreciated by the men involved (Cordier et al., 2016). Prosocial behaviour such as volunteer mentoring (Eisenberg et al., 2007) may increase a participant's feeling of having sufficient time to pursue activities that are personally meaningful and boost their sense of self‐efficacy (Mogilner et al., 2012). Forming an appreciation of the wholeness of another person and their subjective experience (Steele, 2013) strongly aligns with mutuality (Jordan & Stone Center for Developmental Services and Studies, 1986). The theme of mutual growth from the literature offers the participant a broadening of perspective and a diversity of experience via their connection.

Mentoring has long been in the public consciousness. Homer (circa 800 BCE), wrote the Odyssey in which each of the themes from the literature on adult voluntary male mentoring are stored (Rose, 1967). The god Adelphi voluntarily became the “Mentor” character and intentionally formed a relationship with the inexperienced prince. They began a journey, which entailed development and growth. These themes, synthesised from the literature, have implications for connection by men to other men.

A voluntary adult male relationship which is intentional implies that it is able to be managed by the participants soon after the connection is formed. This minimises the community resources going forward to maintain that relationship. Ongoing meetings between men, in which a meaningful connection has been formed, may reduce loneliness (Kearns et al., 2015). Furthermore, the journey theme implies the connection is transitional and finite, possibly reducing a barrier to participation based on the uncertainty of the commitment. Lastly, forming connection with other men, even when they are from different generations or backgrounds, is beneficial to participants' well‐being (Huang et al., 2019).

Some limitations of this review are that it focused on voluntary mentorship, thereby excluding many articles with rich narratives of connection from areas in which individual or organisational compliance may have been present. This exclusion encompassed the disability sector that at times contained insightful mentoring findings. The scholarly articles included in this review were not evaluated for their quality, and some included articles did not differentiate gender in their reporting. Additionally, some of the included articles examined community‐facilitated programs which may have influenced the participants language and the reported narratives.

The themes from the literature present male connection as an affirming and caring interaction, manageable in terms of commitment, and personally beneficial (Seeman et al., 2020). Connecting with others, not only alleviates loneliness but improves the well‐being of both participants (Theurer et al., 2020). Men connecting with other men can reverse the growing number of reported lonely in western society. This article has presented themes from voluntary male mentors which imply the capability is present in our communities, and the benefits are two‐way in a one‐to‐one relationship.

## 5 CONCLUSION

The aim of this scoping review was to present an evidenced narrative of mentoring applicable for utilisation by community participants in modern western societies. Critically examining the narratives of the literature on the subject of adult male voluntary mentoring has provided themes which may inform male connection. Whilst potentially useful to community participants, this review has revealed a lack of research on the lived experience of the men who specifically offer connection to other men through male mentoring. The potential for men to voluntarily connect with others presents an opportunity in communities to ameliorate loneliness and improve the well‐being of men. More research on this area may reveal critical information to increase participation by men in this area of one‐to‐one connection.

## AUTHOR CONTRIBUTIONS

All four authors have participated in the planning of the study and data analysis. The first author performed the data collection and the interviews. The first author drafted the manuscript, and all authors contributed to the final manuscript.

## FUNDING INFORMATION

No financial or material support has been provided in the production of this article.

## CONFLICT OF INTEREST

The authors are not aware of any conflicts of interest.

## Data Availability

Data sharing not applicable to this article as no datasets were generated or analysed during the current study.
